# Cardiorenal protective effects of dapagliflozin combined with valsartan in patients with type 2 diabetes mellitus and hypertension: a retrospective cohort study

**DOI:** 10.3389/fendo.2026.1804226

**Published:** 2026-04-24

**Authors:** Zhengrong Zhou, Kaizheng Gong

**Affiliations:** 1 Medical Department, Yangzhou University, Yangzhou, Jiangsu, China; 2 Department of Cardiology, Affiliated Hospital of Yangzhou University, Yangzhou, Jiangsu, China

**Keywords:** cardiorenal protection, dapagliflozin, glycemic variability, NT-ProBNP, valsartan

## Abstract

**Background:**

Hypertension and Type 2 Diabetes Mellitus (T2DM) interact to increase cardiorenal risk. This study evaluated the cardiorenal efficacy of dapagliflozin combined with valsartan in patients with coexisting T2DM and hypertension.

**Methods:**

This retrospective cohort study included 245 patients admitted between January 2023 and December 2024. Propensity score matching yielded 102 patients in the Monotherapy Group (valsartan 80 mg/day) and 102 in the Combination Group (valsartan 80 mg/day + dapagliflozin 10 mg/day). Outcomes assessed at baseline and 24 weeks included blood pressure, glycemic parameters (FPG, 2hPG, GluCV, PGE, LAGE, MAGE), inflammatory and cardiac biomarkers (hs-CRP, TNF-α, IL-6, IL-33, sST2, sICAM-1), echocardiographic measures (LVEF, LVEDD, LVESD, LAD, IVSd), NT-proBNP, renal function (eGFR, UACR, SCr, BUN), and fibrosis markers (PIIINP, C-IV, LN, TGF-β1).

**Results:**

At 24 weeks, the Combination Group showed greater reductions in systolic (134.86 ± 6.37 vs. 139.68 ± 6.82 mmHg) and diastolic blood pressure (83.12 ± 4.58 vs. 86.29 ± 4.79 mmHg), FPG (6.79 ± 1.02 vs. 7.58 ± 1.10 mmol/L), 2hPG (9.71 ± 1.68 vs. 11.24 ± 1.84 mmol/L), and glycemic variability indices (all P < 0.001). Inflammatory and cardiac biomarkers declined more markedly (P < 0.05). Significant improvements were also observed in LVEF (60.12 ± 4.68% vs. 56.75 ± 4.96%), NT-proBNP, eGFR, UACR, and fibrosis markers (all P < 0.05). Multivariate analysis showed combination therapy independently predicted better outcomes (OR = 2.417, 95% CI: 1.315–4.443, P = 0.004).

**Conclusion:**

The addition of dapagliflozin to valsartan was associated with superior cardiorenal outcomes compared to valsartan monotherapy in patients with T2DM and hypertension, as evidenced by enhanced improvements in blood pressure, glycemic control, inflammation, cardiac function, and renal parameters.

## 1 Introduction

Two of the most common chronic non-communicable diseases across the globe, as well as often occurring in the same individual, are type 2 diabetes mellitus (T2DM) and hypertension (1–3). The two conditions are not accidental because the pathogenic pathways are common, such as insulin resistance, chronic low-grade inflammation, and endothelial dysfunction (4). Their interaction with each other is synergistic, and it has profound outcomes in terms of creating microvascular and macrovascular complications, which have a heavy burden on cardiovascular and renal diseases (5, 6).

Consequently, patients with concomitant T2DM and hypertension constitute a high-risk population, necessitating aggressive and multifaceted management strategies aimed not only at glycemic and blood pressure control but also at direct organ protection.

Current management guidelines emphasize stringent control of both hyperglycemia and hypertension to mitigate long-term risks (7, 8). However, conventional antihyperglycemic and antihypertensive therapies often fail to fully address the intertwined pathophysiological mechanisms that drive progressive cardiac and renal damage. This gap highlights the critical need for therapeutic agents that offer pleiotropic benefits beyond the primary metabolic and hemodynamic effects, specifically targeting the underlying inflammatory, fibrotic, and stress pathways implicated in cardiorenal deterioration (9–11).

Sodium-glucose cotransporter 2 (SGLT2) inhibitors and angiotensin II receptor blockers (ARBs) are some of the promising candidates in this context (12). ARBs e.g. valsartan have been studied extensively with regard to antihypertensive and renoprotective activities that occur via the renin-angiotensin-aldosterone system (RAAS) blockage (13). Dapagliflozin is an SGLT2 inhibitor, which is associated with cardiorenal benefits in diabetes mellitus type 2 (T2DM) patients (14). They have more mechanisms than glucosuria; they also involve the improvement of hemodynamics, the decrease of inflammatory markers, and immediate influence on cardiac and renal fibrosis.

In addition to glycemic markers, SGLT2 inhibitors have now been included in the guidelines to treat heart failure with reduced and preserved ejection fraction, chronic kidney disease, as well as patients with a known atherosclerotic cardiovascular disease (ASCVD), regardless of their glycemic or hypertensive condition. The theoretical explanation behind the combination of these two types of drugs is strong since the complementary actions of these drugs have been linked to positive outcomes of the heart and kidneys (15, 16).

Valsartan and dapagliflozin have their own advantages, but strong clinical data comparing their effectiveness in a patient group in high risk of developing T2DM and hypertension is not so well-researched. There is an urgent need to explore whether such a combination therapy is associated with superior cardiorenal protection compared to standard RAAS blockade alone, thereby addressing a crucial unmet need in clinical practice. Thus, the purpose of this study was to assess the potential cardiorenal benefits associated with the combination of dapagliflozin and valsartan in patients with T2DM and concomitant hypertension.

## 2 Methods

### 2.1 Study design and participant selection

To assess the cardiorenal protective benefits of dapagliflozin with valsartan in patients with type 2 diabetes mellitus (T2DM) and concurrent hypertension, this retrospective cohort study was carried out at our hospital. Our hospital’s Institutional Ethics Committee evaluated and approved the study protocol (Approval No: [20TK214]KS-1079). Because the analysis was retrospective and solely examined anonymized clinical data, informed permission was not required. Every procedure followed the Declaration of Helsinki’s ethical guidelines. A total of 245 individuals with hypertension and type 2 diabetes who were admitted between January 2023 and December 2024 were initially included. To minimize selection bias and potential confounding, propensity score matching (PSM) was subsequently performed using baseline demographic and clinical characteristics, including age, sex, body mass index, duration of hypertension and type 2 diabetes mellitus, New York Heart Association functional class, and relevant comorbidities (**Supplementary Table 1**). A 1:1 nearest-neighbor matching algorithm with a caliper width of 0.2 was applied, yielding 102 patients in each group for the primary analyses. Adult patients (age ≥ 18) with a confirmed diagnosis of type 2 diabetes (T2DM) in accordance with the guidelines for the prevention and treatment of type 2 diabetes (17) and a concurrent diagnosis of hypertension as defined by the guidelines for the management of hypertension (18) met the following inclusion criteria: (1) patients with complete clinical data, including detailed records of treatment regimens, laboratory results, and echocardiographic parameters; and (2) patients who had been on a stable therapeutic regimen for at least 24 weeks prior to data collection. The following were the main exclusion criteria: (1) patients with a history of unstable angina, acute myocardial infarction, or previous cardiac bypass surgery; (2) patients with severe hepatic or renal dysfunction (end-stage renal disease requiring dialysis); (3) patients with active chronic infections, hematological diseases, or malignancies; (4) a history of type 1 diabetes, secondary diabetes, or acute diabetic complications; (5) patients receiving systemic corticosteroids, immunosuppressants, or cytotoxic drugs during the three months prior to the study period; and (7) patients with a diagnosis of heart failure with reduced ejection fraction (HFrEF) or preserved echocardiographic records or clinical documentation.

### 2.2 Therapeutic Regimens and Group Allocation

All patients received standard treatments, including Metformin and Insulin for glycemic control, antihypertensive therapy, lipid-lowering agents, and anti-infective treatment as needed, as well as guidance on physical exercise. Eligible patients (n= 204) were mainly divided into two groups based on the antihypertensive and antidiabetic treatment regimens recorded in the patients’ medical records. Monotherapy Group was comprised of 102 patients who were administered valsartan capsules (Novartis Pharmaceuticals Corporation; Approval No. H20040217 in China; Batch Number: X2859) orally once daily at a dose of 80 mg per administration. The treatment duration was 24 weeks in both groups. Combination Therapy Group: This group included 102 patients. In addition to the monotherapy regimen, dapagliflozin (AstraZeneca Pharmaceuticals LP; Approval No. J20170040 in China; Batch Number: MB2551) was added to their treatment plan. Patients received oral dapagliflozin 10 mg once daily. The treatment duration was 24 weeks. All patients received standard concomitant care, including dietary advice tailored to their conditions and lifestyle modifications aimed at improving overall health outcomes.

### 2.3 Data collection and assessment methodology blood sample collection and processing

A standardized case report form was used to retrieve extensive clinical and laboratory data from the hospital’s digital health record system. Key data points collected at baseline (prior to treatment initiation) and after 24 weeks of treatment included demographic information, medical history, vital signs, laboratory parameters, echocardiographic findings, and records of adverse events.

### 2.4 Blood sample collection and processing

Fasting peripheral venous blood samples were obtained from all participants at baseline and again at the 24-week follow-up visit. Approximately 8–10 mL of blood was collected from the antecubital vein using standard venipuncture procedures. For serum separation, the collected blood was left undisturbed at room temperature for 30 minutes to allow clot formation, followed by centrifugation at 3000 rpm for 15 minutes using a TD4C benchtop low-speed centrifuge (Shanghai Lu Xiangyi Centrifuge Instrument Co., Ltd., China). The resulting serum was carefully transferred into sterile Eppendorf tubes in aliquots and stored at −80 °C in an ultra-low temperature freezer (Thermo Fisher Scientific, USA) until further batch analysis to maintain biomarker integrity.

### 2.5 Assessment of glycemic control and variability

Glycemic control was assessed using several biochemical and monitoring-based indicators. Fasting plasma glucose (FPG) and 2-hour postprandial plasma glucose (2hPG) levels were determined after a standard 75 g oral glucose tolerance test (OGTT). Plasma glucose concentrations were quantified using the hexokinase enzymatic assay on a Cobas c 501 automated clinical chemistry analyzer (Roche Diagnostics, Basel, Switzerland). Glucose fluctuation patterns were further evaluated using data obtained from a continuous glucose monitoring (CGM) device (FreeStyle Libre, Abbott Diabetes Care, USA). Participants wore CGM sensors for at least 72 hours at both assessment points. Several indices reflecting glycemic variability were derived from the CGM recordings, including the glucose coefficient of variation (GluCV), postprandial glucose excursion (PGE), largest amplitude of glycemic excursions (LAGE), and mean amplitude of glycemic excursions (MAGE). Postprandial glucose excursion was calculated as the mean incremental rise in glucose levels following the three principal daily meals according to the formula: [2-hour post-breakfast glucose − pre-breakfast glucose) + (2-hour post-lunch glucose − pre-lunch glucose) + (2-hour post-dinner glucose − pre-dinner glucose)]/3. Both OGTT testing and CGM monitoring were conducted under standardized clinical conditions at our institution at baseline and again after 24 weeks. Participants lacking complete OGTT or CGM datasets were excluded from analyses involving those specific parameters. Missing values were not imputed; instead, analyses were performed using available complete-case observations.

### 2.6 Quantification of inflammatory and cardiac stress biomarkers

Serum concentrations of selected inflammatory mediators and biomarkers related to cardiac stress were measured using enzyme-linked immunosorbent assay (ELISA) techniques. High-sensitivity C-reactive protein (hs-CRP), tumor necrosis factor-alpha (TNF-α), interleukin-6 (IL-6), and interleukin-33 (IL-33) were quantified with commercially available high-sensitivity ELISA kits obtained from Abcam (USA) (catalog numbers: ab260058 for hs-CRP, ab213467 for TNF-α, ab246838 for IL-6, and ab118503 for IL-33). Levels of soluble suppression of tumorigenicity-2 (sST2), a biomarker associated with myocardial stress and fibrotic remodeling, were also determined using a dedicated ELISA kit (Abcam, USA; ab141210). In addition, serum soluble intercellular adhesion molecule-1 (sICAM-1), an indicator of endothelial activation and vascular dysfunction, was measured using a corresponding ELISA kit (Abcam, USA; ab174445). All assays were carried out in duplicate following the protocols provided by the manufacturer to ensure analytical reliability and reproducibility.

### 2.7 Evaluation of cardiac function

Cardiac morphology and functional performance were evaluated using noninvasive two-dimensional transthoracic echocardiography. Examinations were performed with a Vivid E9 ultrasound platform (GE Healthcare, USA) fitted with an M5S phased-array probe. Image acquisition and measurements followed the standardized recommendations of the American Society of Echocardiography. Key echocardiographic indices included left ventricular ejection fraction (LVEF), determined using the biplane Simpson method, left ventricular end-diastolic diameter (LVEDD), left ventricular end-systolic diameter (LVESD), left atrial diameter (LAD), and interventricular septal thickness measured at end-diastole (IVSd). Moreover, circulating levels of N-terminal pro–B-type natriuretic peptide (NT-proBNP), an established biomarker associated with cardiac dysfunction and heart failure, were quantified using an electrochemiluminescence immunoassay performed on a Cobas e 411 analyzer (Roche Diagnostics, Basel, Switzerland) with the manufacturer’s original reagent kits.

### 2.8 Assessment of renal function and fibrosis markers

Renal function was assessed through estimation of the glomerular filtration rate (eGFR) calculated using the Chronic Kidney Disease Epidemiology Collaboration (CKD-EPI) formula, which incorporates serum creatinine concentration together with demographic variables including age, sex, and race. Serum creatinine (SCr) and blood urea nitrogen (BUN) concentrations were determined by enzymatic assays performed on a Beckman AU5800 fully automated biochemical analyzer (Beckman Coulter, USA). Renal injury was further evaluated by measuring the urinary albumin-to-creatinine ratio (UACR) using a first-morning spot urine specimen. Urinary albumin levels were quantified via an immunoturbidimetric assay, while urinary creatinine was determined using the Jaffe colorimetric method, both conducted on the Beckman AU5800 system. To further investigate markers associated with renal fibrotic remodeling, several extracellular matrix–related biomarkers were analyzed in serum samples. Procollagen III N-terminal peptide (PIIINP) was quantified using a radioimmunoassay kit (Orion Diagnostica, Finland; catalog number REA-31-101). In addition, serum concentrations of type IV collagen (C-IV), laminin (LN), and transforming growth factor-β1 (TGF-β1) were measured using commercially available enzyme-linked immunosorbent assay kits (Cloud-Clone Corp., USA; catalog numbers SEA571Hu for C-IV, SEA084Hu for LN, and SEA124Hu for TGF-β1).

### 2.9 Safety and adverse event monitoring

Patient safety was monitored throughout the 24-week study period by reviewing clinical notes, laboratory reports, and patient complaints documented in the medical records. Adverse events (AEs) of special interest for the study drugs were systematically recorded, including dry mouth, dizziness, hypotension, angioedema, urinary tract infection, and liver injury.

### 2.10 Assessment of clinical efficacy statistical analysis

The clinical efficacy of the treatment regimens was evaluated at the end of the 24-week study period based on a composite criterion integrating cardiac functional improvement, glycemic control, and symptomatic relief. Treatment outcomes were categorized into three grades: markedly effective, effective, and ineffective. A patient was classified as ‘Markedly Effective’ if their New York Heart Association (NYHA) functional class improved by two grades compared to baseline, their glycosylated hemoglobin (HbA1c) level was reduced to below 6.5%, and their core clinical symptoms were essentially relieved. An outcome was defined as ‘Effective’ if the patient’s NYHA functional class improved by one grade, their HbA1c was reduced to below 7.0%, and their clinical symptoms showed partial relief. The outcome was considered ‘Ineffective’ if there was no improvement (or worsening) in NYHA functional class, no significant reduction in HbA1c level from baseline, and no relief of clinical symptoms. The overall total effective rate for each treatment group was subsequently calculated as the sum of the number of patients classified as ‘Markedly Effective’ and ‘Effective,’ divided by the total number of patients in that group, and then multiplied by 100% to express it as a percentage.

It is acknowledged that this composite efficacy classification is not a validated international endpoint. It is reported as a supplementary clinical summary metric reflecting composite improvement across functional, glycemic, and symptomatic domains and should be interpreted descriptively rather than as a primary outcome measure.

### 2.11 Statistical analysis

All collected study variables were compiled and analyzed using SPSS statistical software (version 29.0; IBM Corp., USA). Continuous variables with a normal distribution are expressed as mean ± standard deviation ( $\bar{\text{x}}$ ± s), and comparisons between groups were conducted using the independent-samples t-test. Categorical variables are reported as number and percentage [n (%)], and differences between groups were evaluated using the chi-square (χ²) test. To assess the independent association between treatment allocation and clinical efficacy, a multivariate logistic regression analysis was performed. The dependent variable was the effective rate, and the model included treatment group (combination therapy vs. monotherapy) as the primary independent variable, with adjustment for potential confounders including age, sex, body mass index, duration of hypertension, duration of type 2 diabetes mellitus, baseline New York Heart Association functional class, and presence of diabetic complications. Results are presented as odds ratios (OR) with 95% confidence intervals (CI). All statistical analyses were performed using two-sided tests, and a P value < 0.05 was considered to indicate statistical significance.

## 3 Results

### 3.1 Baseline demographic and clinical characteristics after propensity score matching

Following propensity score matching, 102 well-matched pairs (n=204) were included in the comparative analysis. The baseline demographic and clinical profiles were similar across the two matched study groups. As detailed in **Table 1**, no significant differences were observed between the Monotherapy and Combination Therapy groups with respect to age, body mass index(BMI), sex distribution, smoking or alcohol use, hyperlipidemia prevalence, heart rate, duration of hypertension or type 2 diabetes mellitus (T2DM), family history of T2DM, occurrence of diabetic complications, or New York Heart Association (NYHA) functional class (all *P* > 0.05). These findings indicate that the groups were well-balanced at baseline, thereby reducing the likelihood of confounding effects in the subsequent comparative analyses.

**Table 1:** Baseline Characteristics After Propensity Score Matching.

Indicators	Monotherapy Group (n=102)	Combination Group (n=102)	t/χ²	*P*
Age (years)	58.34 ± 6.74	57.91 ± 7.02	0.441	0.660
BMI (kg/m²)	26.47 ± 3.21	26.83 ± 3.08	0.81	0.419
Sex [n (%)]			0.02	0.888
Male	55 (53.92%)	54 (52.94%)		
Female	47 (46.08%)	48 (47.06%)		
History of smoking [n (%)]	36 (35.29%)	38 (37.25%)	0.085	0.771
History of alcohol [n (%)]	29 (28.43%)	31 (30.39%)	0.094	0.759
High blood lipids [n (%)]	78 (76.47%)	75 (73.53%)	0.235	0.628
HR (beats/min)	78.62 ± 7.39	77.84 ± 6.92	0.775	0.439
Duration of Hypertension (years)	6.73 ± 2.14	6.91 ± 2.37	0.569	0.570
Duration of T2DM (years)	7.48 ± 2.62	7.26 ± 2.71	0.589	0.556
Family history of T2DM [n (%)]	39 (38.24%)	42 (41.18%)	0.184	0.668
Diabetic retinopathy [n (%)]	23 (22.55%)	24 (23.53%)	0.028	0.868
Diabetic neuropathy [n (%)]	27 (26.47%)	28 (27.45%)	0.025	0.875
NYHA Classification [n (%)]			0.084	0.772
Grade II	63 (61.76%)	65 (63.73%)		
Grade III	39 (38.24%)	37 (36.27%)		

BMI, Body Mass Index; T2DM, Type 2 Diabetes Mellitus; NYHA, New York Heart Association. Patients with a prior diagnosis of heart failure (HFrEF or HFpEF) were excluded from the study cohort.

### 3.2 Comparison of blood pressure control

After 24 weeks of treatment, both groups exhibited decreases in systolic blood pressure (SBP), diastolic blood pressure (DBP), and mean arterial pressure (MAP). Notably, the reductions in these parameters were significantly more pronounced in the Combination Therapy Group compared with the Monotherapy Group (all *P* < 0.001; **Table 2**). These findings indicate that adding dapagliflozin to valsartan may enhance blood pressure-lowering effects.

**Table 2:** Comparison of blood pressure between the two groups.

Indicators	Monotherapy Group (n=102)	Combination Group (n=102)	t	*P*
SBP (mmHg)				
Before treatment	152.36 ± 8.46	152.98 ± 8.82	0.512	0.609
After treatment	139.68 ± 6.82	134.86 ± 6.37	5.216	< 0.001
DBP (mmHg)				
Before treatment	93.52 ± 5.66	94.03 ± 5.81	0.634	0.527
After treatment	86.29 ± 4.79	83.12 ± 4.58	4.830	< 0.001
MAP (mmHg)				
Before treatment	113.13 ± 5.86	113.68 ± 6.14	0.655	0.513
After treatment	104.09 ± 4.88	100.18 ± 4.69	5.828	< 0.001

SBP, Systolic Blood Pressure; DBP, Diastolic Blood Pressure; MAP, Mean Arterial Pressure.

### 3.3 Comparison of glycemic control and variability parameters

Both treatment groups experienced improvements in glycemic control following the intervention; however, the Combination Therapy Group exhibited markedly better results. After 24 weeks, significant differences between the groups were noted for fasting plasma glucose (FPG), 2-hour postprandial glucose (2hPG), glucose coefficient of variation (GluCV), postprandial glucose excursion (PGE), largest amplitude of glycemic excursions (LAGE), and mean amplitude of glycemic excursions (MAGE), all favoring the combination regimen (all *P* < 0.001; **Table 3**). These results indicate that combining therapies not only lowers average glucose levels but also effectively attenuates glycemic variability, an important factor in the development of diabetic complications.

**Table 3:** Comparison of glycemic parameters between the two groups.

Indicators	Monotherapy Group (n=102)	Combination Group (n=102)	t	*P*
FPG (mmol/L)				
Before treatment	8.92 ± 1.35	9.04 ± 1.29	0.673	0.502
After treatment	7.58 ± 1.10	6.79 ± 1.02	5.302	< 0.001
2hPG (mmol/L)				
Before treatment	13.47 ± 2.16	13.54 ± 2.11	0.222	0.824
After treatment	11.24 ± 1.84	9.71 ± 1.68	6.200	< 0.001
GluCV (%)				
Before treatment	28.34 ± 4.17	28.02 ± 4.31	0.549	0.584
After treatment	24.09 ± 3.79	21.01 ± 3.42	6.087	< 0.001
PGE (mmol/L)				
Before treatment	5.73 ± 1.12	5.66 ± 1.08	0.464	0.643
After treatment	4.89 ± 0.96	4.11 ± 0.86	6.139	< 0.001
LAGE (mmol/L)				
Before treatment	7.56 ± 1.43	7.47 ± 1.39	0.446	0.656
After treatment	6.44 ± 1.21	5.59 ± 1.09	5.253	< 0.001
MAGE (mmol/L)				
Before treatment	4.82 ± 0.96	4.74 ± 0.93	0.628	0.531
After treatment	4.09 ± 0.83	3.49 ± 0.79	5.360	< 0.001

FPG, Fasting Plasma Glucose; 2hPG, 2-hour Postprandial Glucose; GluCV, Glucose Coefficient of Variation; PGE, Postprandial Glucose Excursion; LAGE, Largest Amplitude of Glycemic Excursions; MAGE, Mean Amplitude of Glycemic Excursions.

### 3.4 Comparison of inflammatory, cardiac stress, and endothelial biomarkers

Post-treatment, both groups exhibited decreases in serum levels of hs-CRP, TNF-α, IL-33, IL-6, and sST2, with the Combination Therapy Group showing significantly larger reductions (all *P* < 0.05; **Table 4**). In contrast, changes in sICAM-1 did not differ significantly between the groups (*P* = 0.108). These findings suggest that the combination therapy was associated with enhanced reductions in inflammatory and cardioprotective markers, which may contribute to the observed clinical differences.

**Table 4:** Comparison of inflammatory, myocardial stress and endothelial function biomarkers between the two groups.

Indicators	Monotherapy Group (n=102)	Combination Group (n=102)	t	*P*
hs-CRP (mg/L)				
Before treatment	4.32 ± 1.28	4.39 ± 1.22	0.220	0.826
After treatment	3.68 ± 1.04	3.16 ± 1.06	3.539	< 0.001
TNF-α (pg/mL)				
Before treatment	18.76 ± 4.23	18.94 ± 4.17	0.315	0.753
After treatment	16.93 ± 3.52	15.38 ± 3.74	3.053	0.003
IL-33 (ng/mL)				
Before treatment	0.86 ± 0.23	0.84 ± 0.22	0.708	0.480
After treatment	0.79 ± 0.18	0.71 ± 0.19	3.030	0.003
IL-6 (ng/L)				
Before treatment	7.53 ± 2.14	7.59 ± 2.09	0.214	0.831
After treatment	6.79 ± 1.83	6.14 ± 1.82	2.552	0.011
sST2 (μg/L)				
Before treatment	28.47 ± 6.39	29.03 ± 6.48	0.619	0.537
After treatment	26.58 ± 5.12	24.42 ± 5.31	2.956	0.003
sICAM-1 (μg/L)				
Before treatment	311.46 ± 48.27	309.12 ± 47.86	0.348	0.728
After treatment	286.25 ± 43.41	276.59 ± 42.07	1.613	0.108

hs-CRP, high-sensitivity C-Reactive Protein; TNF-α, Tumor Necrosis Factor-alpha; IL-6, Interleukin-6; IL-33, Interleukin-33; sST2, soluble Suppression of Tumorigenicity-2; sICAM-1, soluble Intercellular Adhesion Molecule-1.

### 3.5 Comparison of echocardiographic parameters and NT-proBNP

**Figure 1:**
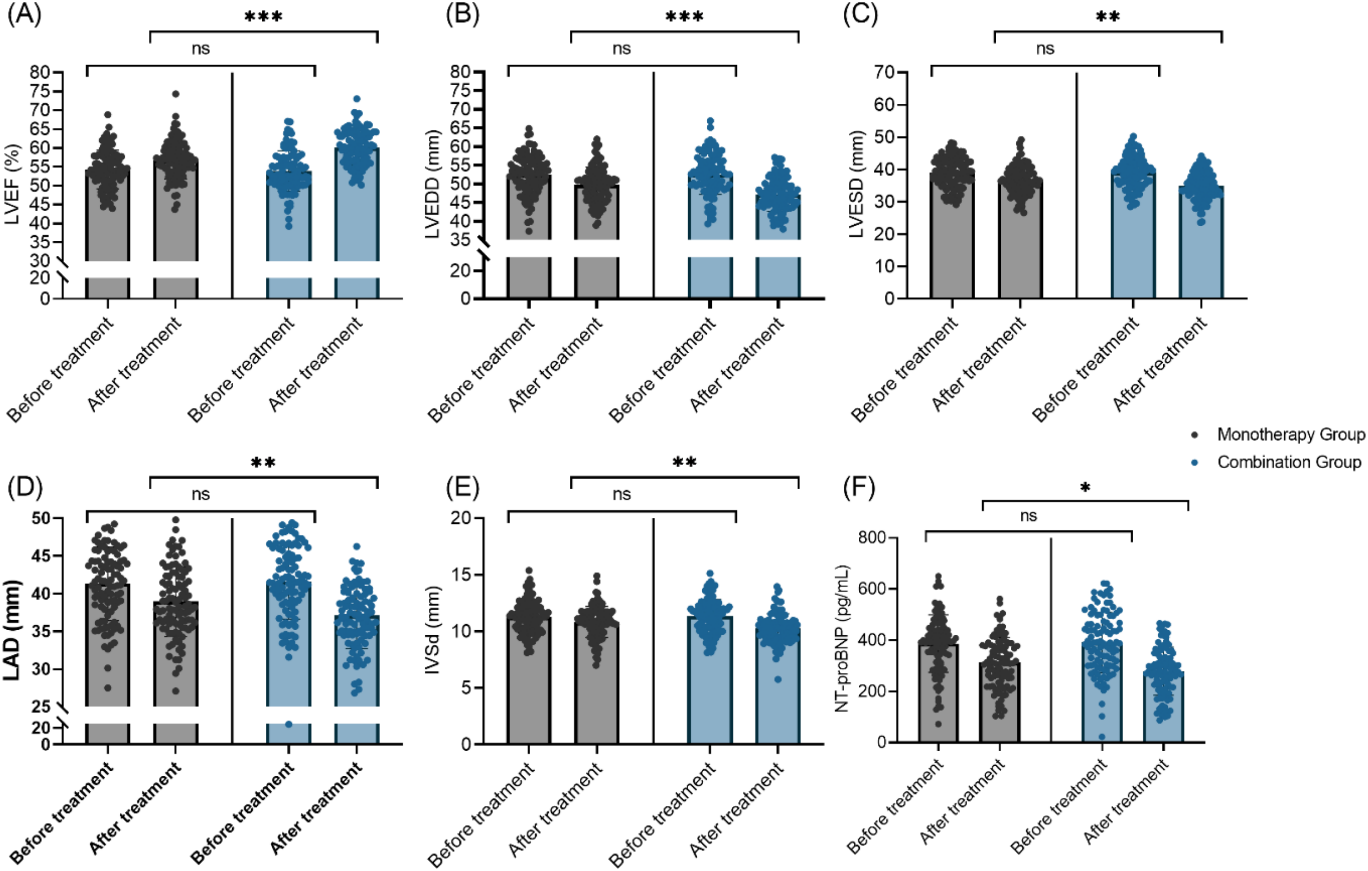
Comparison of echocardiographic cardiac function parameters between the two groups. **(A)** LVEF (%); **(B)** LVEDD (mm); **(C)** LVESD (mm); **(D)** LAD (mm); **(E)** IVSd (mm); **(F)** NT-proBNP (pg/mL). LVEF, Left Ventricular Ejection Fraction; LVEDD, Left Ventricular End-Diastolic Diameter; LVESD, Left Ventricular End-Systolic Diameter; LAD, Left Atrial Diameter; IVSd, Interventricular Septal thickness at diastole; NT-proBNP, N-terminal pro-Brain Natriuretic Peptide. Ns: No significant difference; *P < 0.05; **P < 0.01; ***P < 0.001.

Following treatment, comparison of echocardiographic indices and NT-proBNP levels between the Monotherapy and Combination Therapy groups revealed significant post-treatment differences. Left ventricular ejection fraction (LVEF) improved in both groups, with the Combination Therapy Group demonstrating a greater enhancement (Monotherapy: 54.28 ± 5.14% → 56.75 ± 4.96%; Combination: 53.91 ± 5.37% → 60.12 ± 4.68%; t = 4.992, *P*< 0.001). Likewise, left ventricular end-diastolic diameter (LVEDD), left ventricular end-systolic diameter (LVESD), left atrial diameter (LAD), and interventricular septal thickness at diastole (IVSd) all decreased significantly after treatment in both groups, with more pronounced reductions observed in the combination regimen (LVEDD: t = 3.960, *P*< 0.001; LVESD: t = 3.064, *P* = 0.002; LAD: t = 2.869, *P* = 0.005; IVSd: t = 3.055, *P*= 0.003). Serum NT-proBNP concentrations also declined in both groups, with the Combination Therapy Group showing a larger decrease (t = 2.508, *P* = 0.013). No significant differences were detected between the groups in any parameter at baseline. Collectively, these findings indicate that the addition of dapagliflozin to valsartan may result in superior improvements in cardiac function and NT-proBNP levels compared with monotherapy, highlighting its potential advantage in the management of heart failure (**Figure 1**).

### 3.6 Comparison of serum fibrosis and renal function biomarkers

**Figure 2:**
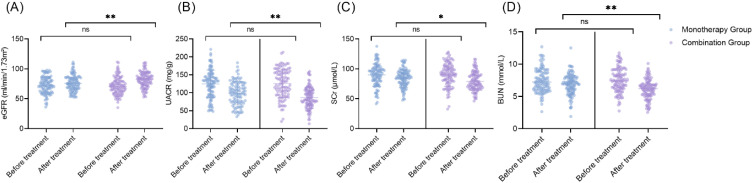
Comparison of renal function parameters between the two groups. **(A)** eGFR (ml/min/1.73m²); **(B)** UACR (mg/g); **(C)** SCr (μmol/L); **(D)** BUN (mmol/L). eGFR, estimated Glomerular Filtration Rate; UACR, Urinary Albumin-to-Creatinine Ratio; SCr, Serum Creatinine; BUN, Blood Urea Nitrogen. Ns: No significant difference; *P < 0.05; **P < 0.01.

After 24 weeks of treatment, the Combination Therapy Group exhibited larger declines in serum markers of fibrosis, including PIIINP, type IV collagen (C-IV), laminin (LN), and transforming growth factor-β1 (TGF-β1), compared with the Monotherapy Group (all *P* < 0.05; **Table 5**). Concerning renal function, estimated glomerular filtration rate (eGFR) increased significantly in both groups, with the Combination Therapy Group showing a more pronounced improvement (t = 2.898, *P* =0.004) (**Figure 2**). Urinary albumin-to-creatinine ratio (UACR) and serum creatinine (SCr) both decreased in both cohorts, with greater reductions observed in the combination regimen (UACR: t =2.781, *P* = 0.006; SCr: t = 2.390, *P* = 0.018). Blood urea nitrogen (BUN) levels also declined significantly across both groups, with the combination therapy yielding a more substantial decrease (t =3.226, *P*= 0.001). No significant differences in renal parameters were detected between the groups at baseline. Overall, these results indicate that combining dapagliflozin with valsartan is associated with enhanced reductions in fibrotic biomarkers and superior improvements in renal function compared with monotherapy.

**Table 5:** Comparison of fibrosis markers between the two groups.

Indicators	Monotherapy Group (n=102)	Combination Group (n=102)	t	*P*
PIIINP (ng/mL)				
Before treatment	49.48 ± 10.63	48.62 ± 10.72	0.573	0.567
After treatment	42.25 ± 7.48	39.14 ± 7.42	2.984	0.003
C-IV (ng/mL)				
Before treatment	70.31 ± 10.26	70.38 ± 10.88	0.052	0.958
After treatment	59.12 ± 10.27	55.38 ± 10.19	2.612	0.010
LN (ng/mL)				
Before treatment	15.48 ± 3.49	15.31 ± 3.27	0.358	0.720
After treatment	10.42 ± 2.34	9.29 ± 2.19	3.573	< 0.001
TGF-β1 (ng/mL)				
Before treatment	32.48 ± 4.19	32.97 ± 4.12	0.831	0.407
After treatment	29.97 ± 3.58	27.13 ± 3.74	5.528	< 0.001

PIIINP, Procollagen III N-terminal peptide; C-IV, Type IV Collagen; LN, Laminin; TGF-β1, Transforming Growth Factor-beta 1.

### 3.7 Adverse cardiovascular events

The overall frequency of adverse events was similar between the two treatment groups (35.29% vs. 23.53%, *P*= 0.065; **Table 6**). Although the occurrence of urinary tract infections was slightly higher in the Combination Therapy Group (6.86% vs. 2.94%), the safety profile of combination therapy was generally comparable to that of monotherapy. Urinary tract infections are a known, manageable side effect associated with SGLT2 inhibitors.

**Table 6:** Comparison of adverse cardiovascular events between the two groups [n (%)].

Indicators	Monotherapy Group (n=102)	Combination Group (n=102)	χ²	*P*
Dry mouth	6 (5.88%)	9 (8.82%)		
Dizziness	8 (7.84%)	9 (8.82%)		
Hypotension	4 (3.92%)	7 (6.86%)		
Angioedema	1 (0.98%)	1 (0.98%)		
Urinary tract infection	3 (2.94%)	7 (6.86%)		
Liver injury	2 (1.96%)	3 (2.94%)		
Total incidence	24 (23.53%)	36 (35.29%)	3.400	0.065

### 3.8 Clinical efficacy

**Figure 3:**
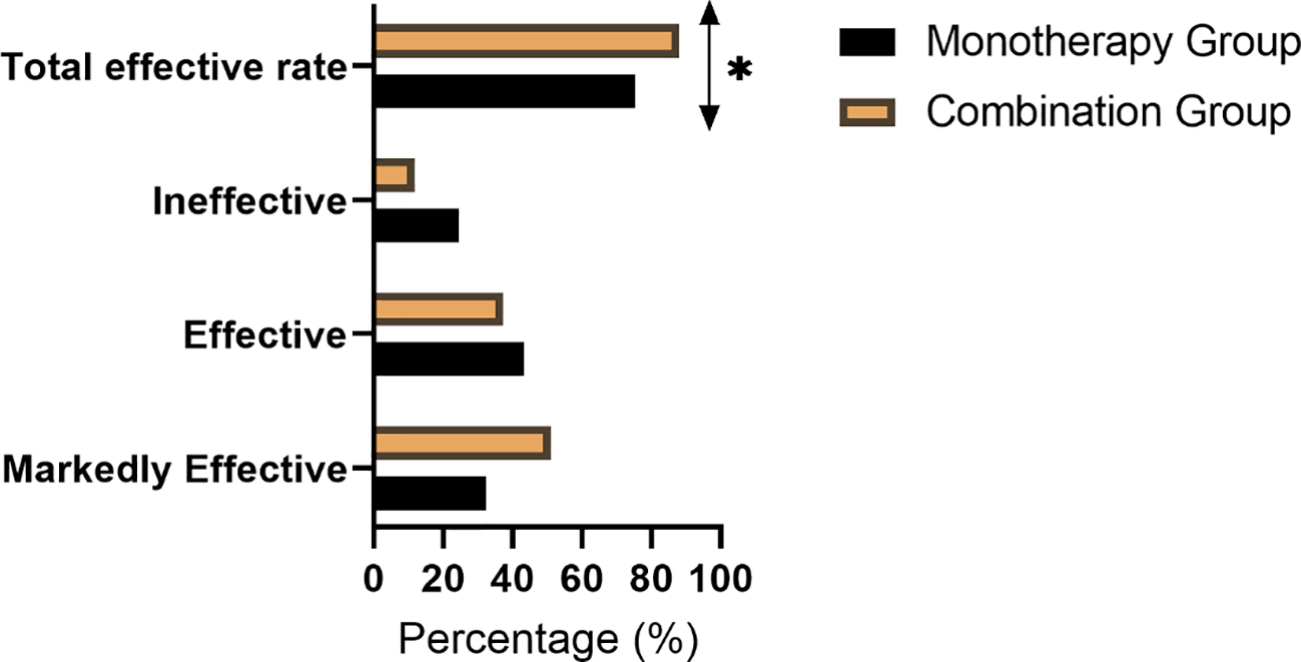
Comparison of clinical efficacy between the two groups [n (%)]. *P < 0.01.

The total effective rate was significantly greater in the Combination Therapy Group (88.24%) compared with the Monotherapy Group (75.49%, *P* = 0.018; **Figure 3**). Additionally, a higher proportion of patients in the combination regimen were classified as “markedly effective” (50.98% vs. 32.35%). These findings support the conclusion that dapagliflozin combined with valsartan provides superior overall clinical efficacy in patients with type 2 diabetes mellitus and hypertension (**Figure 3**).

### 3.9 Multivariate regression analysis of factors associated with clinical efficacy

To further evaluate the independent association between treatment allocation and clinical efficacy while adjusting for potential confounders, a multivariate logistic regression analysis was performed. The dependent variable was the total effective rate (effective vs. ineffective), and the model included treatment group, age, sex, body mass index, duration of hypertension, duration of type 2 diabetes mellitus, baseline NYHA functional class, and presence of diabetic complications. As shown in **Table 7**, combination therapy remained independently associated with a higher likelihood of achieving an effective clinical outcome after adjusting for these covariates (OR = 2.417, 95% CI: 1.315–4.443, *P* = 0.004). No other variables in the model reached statistical significance, including age, sex, BMI, duration of hypertension or diabetes, NYHA class, or diabetic complications (all *P* > 0.05). These findings indicate that the superior clinical efficacy observed with the combination regimen is independent of baseline demographic and clinical characteristics.

**Table 7:** Multivariate logistic regression analysis of factors associated with effective rate.

Variables	β	SE	Wald χ²	OR (95% CI)	*P*
Combination therapy (vs. monotherapy)	0.882	0.310	8.088	2.417 (1.315–4.443)	0.004
Age (per year)	-0.035	0.022	2.506	0.966 (0.924–1.008)	0.113
Sex (male vs. female)	-0.234	0.304	0.591	0.791 (0.435–1.440)	0.442
BMI (per kg/m²)	0.021	0.045	0.218	1.021 (0.936–1.116)	0.641
Duration of hypertension (per year)	-0.008	0.059	0.019	0.992 (0.884–1.113)	0.892
Duration of T2DM (per year)	-0.091	0.050	3.322	0.913 (0.828–1.006)	0.068
NYHA class (III vs. II)	-0.359	0.313	1.316	0.698 (0.378–1.290)	0.251
Diabetic complications (present vs. absent)	-0.468	0.319	2.151	0.626 (0.335–1.171)	0.143

BMI, body mass index; T2DM, type 2 diabetes mellitus; NYHA, New York Heart Association; OR, odds ratio; CI, confidence interval; β, regression coefficient; SE, standard error.

Dependent variable: total effective rate (effective = markedly effective + effective, coded as 1; ineffective coded as 0).

## 4 Discussion

This retrospective cohort study provides a comparative evaluation of the cardiorenal effects of valsartan monotherapy and its combination with dapagliflozin in patients with T2DM and hypertension. These findings suggest that the addition of dapagliflozin to a valsartan-based regimen was associated with more pronounced improvements across a spectrum of cardiometabolic, inflammatory, cardiac functional, and renal parameters over a 24-week period.

In terms of blood pressure control, combination therapy was associated with greater reductions in systolic, diastolic, and mean arterial pressures than monotherapy. This observation aligns with the pharmacodynamic profiles of both agents (19). Valsartan, an angiotensin II receptor antagonist, lowers blood pressure primarily by promoting vasodilation and suppressing the renin-angiotensin-aldosterone system (RAAS) (20). Dapagliflozin, a sodium-glucose cotransporter-2 (SGLT2) inhibitor, exerts mild osmotic diuretic and natriuretic effects, which reduce circulating volume and contribute to blood pressure reduction. The enhanced antihypertensive effect observed with the combination therapy in this study aligns with prior findings, such as those reported by Seidu et al. (21), where concomitant use of an SGLT2 inhibitor and a RAAS blocker produced superior blood pressure control, likely through complementary modulation of vascular tone and fluid homeostasis.

Regarding glycemic control, combination therapy was associated with greater improvements not only in fasting and postprandial glucose levels but also in indices of glycemic variability, including the glucose coefficient of variation, postprandial excursions, and amplitude of glycemic fluctuations. Both poor glycemic control and elevated glycemic variability are recognized as independent risk factors for microvascular and macrovascular complications in diabetes (22, 23). The mechanism underlying this improvement likely involves the insulin-independent action of dapagliflozin, which promotes urinary glucose excretion, thereby lowering plasma glucose levels and potentially flattening glucose peaks (24, 25). This effect complements standard glucose-lowering therapy in all patients. Lee et al. (26) similarly highlighted the unique capacity of SGLT2 inhibitors to attenuate glycemic fluctuations, which may contribute to their cardiorenal benefits beyond HbA1c reduction.

The assessment of inflammatory and cardiac stress biomarkers revealed more marked reductions in hs-CRP, TNF-α, IL-6, IL-33, and sST2 levels in the combination therapy group. Chronic low-grade inflammation and cardiac stress are pivotal in the progression of diabetic cardiomyopathy and heart failure (5). Valsartan possesses anti-inflammatory properties by blocking angiotensin II-mediated pro-inflammatory signaling, as evidenced by recent studies showing its ability to attenuate inflammatory mediators such as IL-6 and TNF-α (20, 27). Dapagliflozin has been shown to reduce inflammatory cytokines and markers of myocardial stress, possibly via improved metabolic efficiency, reduction in visceral adiposity, and direct effects on cardiac fibroblasts (28). The synergistic reduction in these biomarkers observed in this study suggests that this combination may more effectively attenuate the inflammatory and fibrotic pathways driving cardiorenal disease progression in this population. The lack of a between-group difference in sICAM-1 levels post-treatment may indicate that endothelial activation, as reflected by this specific marker, was similarly modulated by both regimens over the study period.

Combination therapy was associated with more pronounced improvements in cardiac structure and function, as reflected by echocardiographic measurements and NT-proBNP levels. In particular, increases in left ventricular ejection fraction and reductions in left ventricular dimensions, left atrial size, interventricular septal thickness, and NT-proBNP were greater in the combination therapy group compared with monotherapy. These findings are consistent with enhanced reverse cardiac remodeling and reduced cardiac wall stress in the combination group, though causality cannot be established from this observational design. The mechanisms are multifactorial, likely stemming from the combined effects of afterload reduction (valsartan), preload reduction (dapagliflozin-induced diuresis), improved myocardial energetics from a shift in fuel utilization (dapagliflozin), and the aforementioned anti-inflammatory and anti-fibrotic effects (29, 30). This aligns with the outcomes of major cardiovascular outcome trials for SGLT2 inhibitors, which have consistently shown benefits in heart failure hospitalization and cardiac structure/function, particularly when used in conjunction with foundational therapies such as RAAS inhibitors (31, 32).

Combination therapy also demonstrated enhanced renal protective effects. The Combination Therapy Group exhibited larger increases in estimated glomerular filtration rate (eGFR) and more substantial decreases in urinary albumin-to-creatinine ratio (UACR), serum creatinine, and blood urea nitrogen (BUN) compared with monotherapy. In parallel, circulating markers of renal fibrosis, including PIIINP, type IV collagen, laminin, and TGF-β1, were reduced to a greater extent in patients receiving the combination regimen. These findings indicate not only functional renal improvement but also potential attenuation of the underlying fibrotic process. The renal benefits of SGLT2 inhibitors are attributed to reduced intraglomerular pressure through tubuloglomerular feedback (33, 34), whereas ARBs, such as valsartan, dilate the efferent arteriole (35). Their combination may offer complementary glomerular hemodynamic effects. Furthermore, the anti-fibrotic properties of both drug classes, as discussed by van Raalte et al. (36) and Chen et al. (37), may inhibit key pathways involved in extracellular matrix deposition, thereby slowing renal fibrosis.

The integrated clinical efficacy assessment, based on a composite of cardiac functional improvement, glycemic control, and symptom relief, revealed a higher total effective rate in the combination therapy group, with a greater proportion of patients achieving a “markedly effective” outcome. This holistic metric reflects the convergent benefits observed across individual physiological domains, translating into comprehensive patient management.

The findings of this analysis have considerable clinical relevance. Patients with T2DM and hypertension represent a high-risk population for accelerated cardiorenal diseases. The observed additive and potentially synergistic benefits of combining dapagliflozin with valsartan on blood pressure control, glycemic variability, inflammatory markers, cardiac remodeling, and renal function indicate that this therapeutic approach may be effective for comprehensive risk reduction. Such effects could contribute to slowing the progression of subclinical cardiac dysfunction and early renal impairment, thereby helping to prevent the development of overt heart failure and chronic kidney disease. The overall safety profile was similar between the groups; although urinary tract infections were slightly more common in the combination therapy group, they remained manageable and consistent with the known adverse effects of SGLT2 inhibitors.

This study has limitations that should be considered. Its retrospective, single-center design may introduce selection bias and unmeasured confounding variables. Although baseline characteristics were comparable between the groups, residual confounding cannot be excluded, as patients receiving dapagliflozin in combination with valsartan may differ in unmeasured factors such as cardiovascular risk profile, clinician prescribing behavior, or socioeconomic status. Importantly, while the sample size was sufficient to detect several between-group differences, it may be underpowered for rare adverse events. The 24-week follow-up period is relatively short for evaluating long-term cardiorenal outcomes or hard endpoints such as cardiovascular mortality or progression to end-stage renal disease. Although echocardiographic assessments were standardized, these measurements remain operator-dependent and subject to variability. Additionally, all participants were on metformin and insulin therapy, which may limit generalizability to patients on other treatment regimens. Finally, the composite clinical efficacy classification applied in this study has not been validated internationally and should be interpreted descriptively.

Future research should prioritize prospective, randomized controlled trials with larger cohorts and extended follow-up periods to confirm these findings and establish causality. Investigations into the molecular mechanisms underlying the observed synergistic effects, particularly regarding fibrosis and inflammation, would provide mechanistic insight. Furthermore, studies evaluating the cost-effectiveness and long-term impact of major adverse cardiorenal events in real-world populations are warranted to define the role of this combination therapy in routine clinical practice.

## 5 Conclusion

In this 24-week retrospective cohort study involving 245 patients with type 2 diabetes mellitus and hypertension, adding dapagliflozin to valsartan was linked to enhanced overall cardiorenal outcomes compared with valsartan alone. The combination regimen resulted in more pronounced reductions in blood pressure, glycemic control with reduced variability, inflammatory and cardiac stress biomarkers, echocardiographic parameters (including LVEF), renal function (eGFR and UACR), and fibrosis markers, along with a higher overall clinical efficacy rate. The safety profiles were comparable, with a manageable increase in urinary tract infections. These findings support further prospective investigation of the combined use of dapagliflozin and valsartan as a potential therapeutic strategy for comprehensive cardiorenal risk modification in high-risk populations.

## Data Availability

The original contributions presented in the study are included in the article/**Supplementary Material**. Further inquiries can be directed to the corresponding author.
