# Supplementary material for: Cardiorenal protective effects of dapagliflozin combined with valsartan in patients with type 2 diabetes mellitus and hypertension: a retrospective cohort study
Source: Front Endocrinol (Lausanne). 2026 Apr 24;17:1804226. doi: 10.3389/fendo.2026.1804226 (PMC13152797; doi:10.3389/fendo.2026.1804226)
Supplement: Supplementary file 1 [file Table1.docx]

**Table S1 Baseline Characteristics Before Propensity Score Matching**

| **Indicators** | **Monotherapy Group (n=117)** | **Combination Group (n=128)** | **t/χ²** | ***P*** |
| --- | --- | --- | --- | --- |
| Age (years) | 58.34±6.74 | 57.91±7.02 | 0.491 | 0.624 |
| BMI (kg/m²) | 26.47±3.21 | 26.83±3.08 | 0.898 | 0.370 |
| Sex [n (%)] |  |  | 0.055 | 0.814 |
| Male | 63 (53.85%) | 67 (52.34%) |  |  |
| Female | 54 (46.15%) | 61 (47.66%) |  |  |
| History of smoking [n (%)] | 41 (35.04%) | 48 (37.50%) | 0.160 | 0.690 |
| History of alcohol [n (%)] | 33 (28.21%) | 39 (30.47%) | 0.151 | 0.698 |
| High blood lipids [n (%)] | 89 (76.07%) | 94 (73.44%) | 0.224 | 0.636 |
| HR (beats/min) | 78.62±7.39 | 77.84±6.92 | 0.852 | 0.395 |
| Duration of Hypertension (years) | 6.73±2.14 | 6.91±2.37 | 0.618 | 0.537 |
| Duration of T2DM (years) | 7.48±2.62 | 7.26±2.71 | 0.637 | 0.525 |
| Family history of T2DM [n (%)] | 45 (38.46%) | 53 (41.41%) | 0.221 | 0.638 |
| Diabetic retinopathy [n (%)] | 27 (23.08%) | 29 (22.66%) | 0.006 | 0.938 |
| Diabetic neuropathy [n (%)] | 31 (26.50%) | 35 (27.34%) | 0.022 | 0.881 |
| NYHA Classification [n (%)] |  |  | 0.079 | 0.778 |
| Grade II | 72 (61.54%) | 81 (63.28%) |  |  |
| Grade III | 45 (38.46%) | 47 (36.72%) |  |  |

Abbreviations: BMI, Body Mass Index; T2DM, Type 2 Diabetes Mellitus; NYHA, New York Heart Association.

Note: Patients with a prior diagnosis of heart failure (HFrEF or HFpEF) were excluded from the study cohort.
